# Granulomatosis with polyangitis presenting with multiple cranial nerve palsies

**DOI:** 10.1186/1471-2474-14-S1-A11

**Published:** 2013-02-14

**Authors:** Muhammad Kazmi, Mohammed Akil, Rachael Kilding

**Affiliations:** 1 Rheumatology Department, Royal Hallamshire Hospital, Sheffield, UK

## Background

Wegener's granulomatosis/granulomatosis with polyangitis classically affects the upper and lower respiratory tracts as well as renal system and lungs, while cranial nerves and meningeal involvement is rare and present in only 2% to 4% of cases.

## Case presentation

We report the case of localised granulomatosis with polyangitis in a 27 year old Caucasian gentleman who presented with multiple cranial neuropathies. He reported diplopia, hearing loss, dysphagia and dysarthria due to involvement of VI, XIII, IX, X and XII cranial nerves. A cerebral MRI demonstrated pachymeningitis along with mastoid changes and additional diagnostic workup showed elevated CRP and C-ANCA (PR-3) antibodies but did not show any pulmonary infiltrates. Induction treatment with prednisolone, cyclophosphamide and rituximab followed by maintenance MMF resulted in complete resolution of his clinical features within six months.

## Conclusion

This case describes this extremely rare presentation of GPA with multiple cranial palsies as initial manifestation, as only less than 100 similar cases are reported in literature worldwide. This case was a diagnostic challenge as there was no eye/para-nasal sinus/chest or renal involvement.

